# Feeling valued at work: a qualitative exploration of allied health profession support workers

**DOI:** 10.1186/s12913-024-11879-z

**Published:** 2024-11-29

**Authors:** Abigail J. Hall, Victoria A. Goodwin, Lorraine Allchurch, Luke Capon, Vicky Farrell, Oludare Olufunmilayo, Richard Griffin

**Affiliations:** 1 Public Health and Sports Science Department, University of Exeter, Heavitree Road, Exeter, EX1 2LU UK; 2 Dudley Group NHS Foundation Trust, Dudley, DY1 2HQ UK; 3 Bedfordshire, Luton and Milton Keynes AHP Faculty, Twinwoods Health Centre, Milton Road, Bedford, Clapham, MK41 6AT UK; 4 Cornwall Partnership NHS Trust, Bellair Health Office, Alverton Road, Penzance, Cornwall TR18 4TA UK; 5 North East London NHS Foundation Trust, The West Wing, CEME Centre, Marsh Way, Rainham, RM13 8GQ UK; 6 Kings Business School, Kings College London, 30 Aldwych, London, WC2B 4BG UK

**Keywords:** Allied health professions, Support workers, Value, AHP

## Abstract

**Objectives:**

The aim of this study was to explore Allied Health Professions (AHP) support worker perceptions of feeling valued and to understand what factors contribute to this feeling of “value”.

**Design:**

This was a qualitative study with semi-structured interviews undertaken virtually. The data were analysed using a process of thematic analysis in order to gain an in depth understanding of the factors that affect support workers feeling “valued”.

**Setting and participants:**

Twenty-nine AHP support workers were recruited. They had a wide range of characteristics and experience in a variety of different settings and working with different professions. Participants all had experience of working in England and data collection was undertaken during February and March 2024.

**Results:**

A sense of belonging, recognition for their skills and abilities, empowerment within their role, as well as opportunities to develop, were seen as key factors contributing to their feeling of value. Where support workers did not feel valued, they often referred to themselves as “just a support worker” and their skills and abilities were not fully utilised. Where support workers did feel valued, they thrived in their roles and enjoyed the opportunities that the role afforded them. There was significant variation in how valued our support workers felt which had a clear impact on them as people, but also would influence the care that they delivered.

**Conclusion:**

AHP support workers are an integral part of the workforce, occupy diverse roles in healthcare and work collaboratively with registered staff, often prioritising patient care over their own career development and ambition. Recognising, empowering, and including them in teams is crucial for a supportive environment. Acknowledging their skills, providing learning opportunities, and supporting their development is essential for their well-being and fostering inclusivity in healthcare. By valuing and nurturing AHP support workers, we improve patient care and build a stronger healthcare workforce.

**Supplementary Information:**

The online version contains supplementary material available at 10.1186/s12913-024-11879-z.

## Background

The Allied Health Profession (AHP) Support workforce comprises of non-registered, but highly skilled professionals. In the NHS, AHPs encompass a diverse range of fourteen professions, such as Physiotherapy, Occupational Therapy, and Radiography, each involving both registered professionals (e.g., Physiotherapists) and support workers (e.g., Physiotherapy Assistants). Support workers in these roles are frequently titled as ‘Assistant’ or ‘Support Worker,’ which can perpetuate a subordinate identity within the healthcare hierarchy. It is estimated that there are 24,000 AHP Support Workers in England [1], all of whom work in a variety of different settings and roles. In the NHS, roles are structured by ‘bands’ which reflect salary, responsibility, and opportunities for progression. AHP support workers typically fall between Bands 2 and 4, with higher bands involving more specialised and autonomous tasks. For example, Band 2 roles generally include basic support tasks, while Band 4 roles involve assisting with assessments and carrying out delegated clinical activities. This banding framework shapes the scope of practice and degree of autonomy for support workers, influencing their experience of value and recognition within healthcare teams.

The NHS Long Term Plan [2] and the NHS Long Term Workforce Plan [1] recognise the importance of these support workers as being vital to deliver effective and safe NHS services. However, there is a massive shortfall, with the NHS Plan estimating that over 204,000 new support workers will be required to meet the demand over the next 15 years [2]. The NHS Plan commits to supporting the development of support workers. The AHP Support Worker Competency, Education and Career Development Framework [3] was introduced to create a clear career development structure and clarify scope of practice. The NHS Long Term Workforce Plan [1] particularly emphasises the importance of promoting routes to professional qualifications for support workers, however, it is evident that there is significant variation and unregulated pathways into support worker roles.

The Cavendish Review [4] explored the use of support workers some ten years ago, recognising the strategic and clinical value of this group of staff, but it was reported that many support workers felt undervalued and taken for granted. Though strategies were put in place to address this, such as the development of the Care Certificate, it is unclear as to whether the recommendations from the report have been integrated in daily practice [5]. It is especially evident that these strategies were not mandated and AHP strategy was designed as there was evidence that there had not been significant change since the Cavendish Review [5]. While there is wide recognition of the importance of support workers, there needs to be consideration as to how they are employed in the NHS [6], while also understanding their challenges and opportunities that they offer.

The recently published Community Rehabilitation [7] and Intermediate Care frameworks [8] highlight the pivotal role that support workers have in ensuring safe and effective rehabilitation in primary care. There is clear emphasis on extending the roles of support workers to enable registered staff to focus on patients with greater complexity, increased autonomy to undertake assessments for “predictable” patients and incorporating the use of digital technologies. Both frameworks suggest an integral role for the AHP support worker in effectively delivering these strategies, however, the suggestion that support workers feel undervalued may have a negative impact on the effectiveness of the integration of these strategies.

Feeling valued at work is fundamental to both individual well-being and organisational effectiveness. Research in organisational psychology indicates that when employees feel valued, they experience higher levels of job satisfaction, increased motivation, and greater engagement, which are essential for both individual performance and team cohesion [9]. In healthcare settings, the importance of feeling valued is amplified, as it directly impacts patient care and the quality of service delivery. Support workers, who often serve in roles that are perceived as less visible, may experience diminished feelings of value, which can lead to higher turnover rates, lower job satisfaction, and reduced engagement in their work [10].

Additionally, the sense of being valued influences mental health outcomes, with studies showing that employees who feel underappreciated are more susceptible to burnout, stress, and disengagement from their work [11]. As support workers often operate in high-demand healthcare environments, acknowledging their contributions is crucial for fostering resilience and maintaining a supportive work environment.

While research has explored the roles of support workers in community and intermediate care [10], no research has focused on the perceptions of the support workers themselves to their role. Thus, the aim of this study was to build on the work of Cavendish and undertake an exploration of what factors affect AHP support workers feeling of being “valued”.

## Methods

### Design

Semi-structured online interviews were undertaken with a range of AHP support workers during February and March 2024. The study has been reported according to the COREQ guidelines (see supplementary material). A stakeholder group comprising AHP support workers from diverse regions and settings across the country was assembled to provide insight and guidance throughout the study. These stakeholders were selected based on their interest and prior involvement in the authors’ earlier work. A virtual workshop was held, where the group engaged in discussions about the study’s design and provided valuable input on ways they could contribute to the data analysis. Their practical experience and perspectives were integral to shaping the study’s approach, ensuring that the research remained relevant and reflective of the support worker role in various healthcare contexts.

### Setting and participants

A purposive sampling strategy was employed with those who consented to capture a variety of different perspectives – including age, levels of qualification, NHS banding, years of experience, location of work, AHP profession supported, gender and ethnicity. The locations of work were categorised according to the seven recognised regions by NHS England. Participants were asked to complete an online expression of interest (EOI) form which made it clear that not everybody would be chosen for an interview.

#### Inclusion criteria

- Support worker with experience of working in the NHS in England.
- Working alongside an AHP profession or multiple professions.
- Consent to take part.

### Recruitment & consent

Participants were sought who worked as support workers working with AHPs. This included any of the AHP professions such as physiotherapy, occupational therapy, speech and language therapy as well as those who work in more generic, cross-profession roles.

A targeted social media campaign using X (formerly known as Twitter) advertised for potential participants to contact the research team to express their interest. The professional bodies/unions were contacted and asked if they could disseminate to their associate (non-registered) members and an email was sent to all regional AHP leads to disseminate to their support worker networks within their regions and organisations.

Participants were informed that they were under no obligation to participate in the interview and they may have withdrawn at any time, without any negative consequence, up until the point where the data was fully anonymised.

A Participant Information Sheet and a link to an EOI form was included in the targeted emails and responses to social media posts. JISC online was used to collate EOIs to take part in the study. JISC Online Surveys (formerly Bristol) online survey tool (https://www.onlinesurveys.ac.uk/) is secure, strict information security standards are followed (ISO27001) and data is processed in compliance with GDPR. Once participants were selected, the online survey was permanently deleted. No downloads were made from the platform.

The EOI included some basic demographic information relating to the sampling strategy (gender, professional background, location of work, years of experience etc.) and to ensure maximum variation, several waves of promotion of the study occurred during recruitment. This included targeted recruitment for particular professions which were under represented.

Support workers who completed the EOI and were selected for interview were then contacted via email, sent the participant information sheet (PIS) (in case they haven’t reviewed the link sent in the introductory email or social media advert) and offered an opportunity to have a telephone or Teams/Zoom call to discuss the study in more detail. A link to book an interview was sent to the participant so they could chose a date/time that suited them best. This link included a consent form and they were asked to return by email before the interview took place.

### Data collection

Interviews were undertaken with a range of participants to explore their experiences of working as an AHP support worker and their perception of feeling valued. The interviews were undertaken by two of the research team (AH and RG) who are both experienced qualitative researchers and have undertaken extensive training in qualitative methodologies. The interviews were undertaken virtually on MS Teams to reduce barriers due to geographical limitations and lasted between 35 and 70 min. We ensured GDPR compliance by utilising Teams’ encrypted recording features, securely transferring and anonymising data post-interview, restricting access to research personnel, and obtaining explicit participant consent for data handling and storage. We aimed to recruit a minimum of 20 participants providing sufficient information power to address the study aims [12]. A topic guide was developed based on the aims and objectives of the research (see supplementary file). Participants were asked the same initial questions, but the questions were worded so that responses are open-ended. This open-endedness allowed the participants to contribute as much detailed information as they desired, and it also allowed the researcher to ask probing questions as a means of follow-up. The questions then varied according to how the interview proceeded, although a guide to potential questions was adhered to (appendix). The topic guide was initially piloted with three support workers, but as no changes were required prior to using it with the remaining participants, this data was included in the analysis. Data collection and initial analysis ran simultaneously. Interviews were recorded via Microsoft Teams and transcribed verbatim using the transcription function that is part of the software. This has been tested to be effective and reliable, but the researcher checked all recordings and transcripts to ensure accuracy. Following transcription, they were anonymised to remove any potentially identifiable information. No follow up interviews were undertaken with participants.

## Analysis

Reflexive thematic analysis [13] was used to make sense of the data as we were interested in determining any themes that were identified. An inductive approach allowed the development of themes that were of relevance to participants rather than testing any existing theory. Familiarisation of the data was undertaken immediately following transcription, followed by a process of open coding. Electronic software, NVivo 11 (QSR International), was used to manage this coding process. From this open coding, initial themes were generated, and core categories identified in a process of selective coding. A coding framework was developed and discussed with the wider research team and the stakeholder group to allow further refinement of the data. A stakeholder engagement meeting, involving a variety of professionals (seven in total) including support worker leads and registered staff, discussed the themes and further refined them.

## Ethics

Ethical approval was gained from King’s College London Research Ethics Committee (reference MRA-23/24-40053).

## Results

Two hundred and thirty-nine participants completed the online EOI form, a total of 47 participants were contacted to arrange an interview from which 29 replied and were interviewed. One person chose an interview time but did not attend and did not reply to a further email follow up. Nobody withdrew from the study. The support workers worked in seven AHP professions, had an average of 10.1 years of experience working as an AHP support worker and there were participants from all seven regions of England.

**Table:** 

	***N*** **= 29 (%)**
**Sex**	
Male	2 (7)
Female	26 (90)
Prefer not to say	1 (3)
**Profession (please note many worked with multiple)**	
Physiotherapy	10 (34)
Occupational therapy	15 (52)
Speech and language therapy	8 (28)
Paramedic	2 (7)
Dietetics	4 (14)
Podiatry	3 (10)
Radiography	2 (7)
**NHS Banding**	
2	2
3	13
4	11
5	1
Unsure / not reported	2
**Location**	
London	3 (10)
East of England	4 (14)
Midlands	4 (14)
South West	6 (21)
South East	6 (21)
North West	2 (7)
North East & Yorkshire	4 (14)
**Years of experience**	
< 1 year	1 (3)
1–5 years	10 (34)
6–10 years	7 (24)
11–15 years	5 (17)
16–20 years	2 (7)
20 + years	4 (14)
**Ethnicity**	
Black or Black British-African	1 (3)
White-British	16 (55)
Mixed – white and black	1 (3)
White – any other background	3 (10)
Not stated	5 (17)
Other	2 (7)

The meaning of being “valued” and what constitutes this was explored with all participants and analysis generated three main themes – recognition, belonging and empowerment.

### Recognition

Recognition was a major theme that support workers felt contributed to their sense of value. This recognition, however, extended beyond completing delegated tasks. Many tasks assigned to support workers require specific knowledge and competencies, allowing them to contribute meaningfully to patient care. One of the clearest factors that affected the support workers feeling of being valued was being recognised for their skills, experience and abilities. Being shown appreciation added belief that they were a valued member of the team, but more than anything it was important that they felt trusted.

#### Appreciation

Being appreciated was reported variably. It was felt that they were appreciated by patients and their relatives often, but the level of appreciation shown by their own teams varied. However, being appreciated was felt to be key to a feeling of value. The methods of being shown appreciation differed – from a simple “thank you” at the end of the day to being given awards.

*when you’ve done something good….just write a little slip and they get put in a box and then at the governance meeting once a month they get read out and then someone will get star of the month. You get a little bottle of wine and chocolate. (Participant 21)*

There were many examples of such awards which were positively viewed and celebrated by our participants. More subtle signs of appreciation were also reported with their skills and expertise being utilised by other members of the team – being asked for advice by registered staff was something that afforded support workers great pride.

Where support workers may not have felt confident in their roles, they relied on being given compliments as a way of feeing appreciated and this increased their confidence in their abilities. Generally our participants reported that their team frequently offered them compliments and recognised how valuable they were to the service. However, several support workers reported that receiving such compliments from their registered colleagues – and managers - was rare, leading to a feeling of inferiority.

*We’re just sidekicks instead of superheroes - our superheroes are the qualified physios*,* OT*,* speech and language*,* and the sidekicks are therapy support workers*,* I don’t get complimented enough (Participant 29).*

This statement reveals the disparity in how support workers perceive their roles versus those of registered staff. The ‘sidekick’ analogy suggests a feeling of being secondary in importance, which reflects the broader theme of inadequate recognition. By identifying registered staff as ‘superheroes,’ the participant underscores a hierarchical dynamic that leaves support workers feeling undervalued.

Such experiences contribute to a diminished sense of identity among support workers, as they feel defined not by their own skills but by their supportive roles. This lack of recognition limits their sense of belonging and professional growth, highlighting the importance of inclusive recognition practices.

Pay was discussed by some of our participants. Those who did discuss pay noted that their skills and expertise was not renumerated appropriately. However, it was evident that pay was rarely the main motivation for a career as a support worker.

Where support workers did not feel valued, they often described themselves as “just as support worker”. When discussed why they referred to themselves, it appeared a historical feeling that they were somehow inferior to registered staff.

*I think unfortunately because they [support workers] have always been referred to as just a support worker….I started doing these support worker meetings to really try and empower them and say you’re not just a support worker (Participant 20)*.

The phrase ‘just a support worker’ captures the internalised impact of hierarchical language on workers’ self-perception. The participant’s initiative to host meetings highlights the importance of re-framing how support workers see themselves. It also suggests that support workers desire more affirmative language to define their roles, which could help reduce feelings of inadequacy and foster a stronger professional identity. These terms influence not only how support workers perceive themselves but also how they are perceived by colleagues and the broader organisation. A shift towards more inclusive and respectful terminology could therefore enhance their sense of value and belonging.

*you work so hard that I think because the support workers are always there*,* the registered members of staff almost take advantage of that …. They don’t always recognise how valuable they are (Participant 20)*.

#### Trust

Being trusted was discussed by many participants – both in terms of being trusted to effectively undertake their role, but also the importance of being able to self-manage their time and diaries.

Support workers described that being delegated tasks could make them feel trusted; however, this often depended on the type of task assigned. Being delegated difficult tasks made them feel they had the appropriate skills and abilities to undertake it, however, being delegated tasks they saw as “menial” meant that they felt that they weren’t trusted to undertake more difficult activities.

*Yeah*,* it’s just feeling trusted because….what am I doing that’s so wrong that you [registered staff] can’t trust me? And yeah*,* and that that it’s quite hurtful actually to be*,* you know*,* to be feel that. You’re not trusted. (Participant 7)*

This participant reflects a recurring sentiment of feeling undermined when tasks are withheld from them. This lack of trust can be interpreted as a form of disempowerment, leading support workers to question their own competencies. It also highlights the emotional toll that such experiences have on workers, who may feel dismissed or underestimated. This sentiment underscores the broader theme of empowerment and how essential it is for support workers to feel trusted with meaningful responsibilities. By delegating tasks that challenge and engage these workers, organisations can foster a stronger sense of trust and value.

Being afforded responsibility for specific areas of the service was seen as being more meaningful than being delegated tasks as this further re-enforced a feeling of being trusted and promoted feelings of self-efficacy. However, not all registered staff were suggested to be good at handing over responsibility which often led to frustration.

Where able, having control over their diaries and tasks was important to our participants. Being micromanaged further reduced the feeling of being trusted.

*We’re worked to the minute*,* you know. Everything’s watched. You know you have how long have you had a break for to go to the toilet and things like that*,* that’s all recorded. (Participant 8)*

#### Scope of practice

The wide scope of practice was highlighted – with examples of participants supporting multiple different AHP professions – often providing interventions for patients with high levels of complexity. However, this was rarely recognised – nor was the scope of practice clearly delineated. Having a clear and defined scope of practice was deemed to be integral to being able to effectively work within the role.

It was evident that there was a lack of consistency between roles and this led to frustration for support workers. Boundaries were not clearly defined which led to uncertainty about what they were able to do. While many people described the presence of competencies, these often varied in different departments or organisations. Some of our participants reported that they didn’t have competencies at all which led to them feeling vulnerable.

*And one of the things that we’re doing as band 4s at the moment is creating our own competencies. Because we feel that it’s inequitable*,* the work we’re given. (Participant 24)*

Participants described various responsibilities, including patient assessments, therapy implementation, and direct patient support, illustrating that these tasks involve much more than simple delegation. For instance, some support workers assisted in patient mobility assessments, a task requiring understanding of basic anatomy, mobility limitations, and safety protocols. Another participant described supporting speech and language therapy sessions, which involved applying learned techniques to help patients with exercises under the supervision of a licensed therapist. These tasks not only demand technical skills but also an understanding of patient care and therapeutic practices.

### Belonging

In discussions around belonging, many support workers highlighted the importance of having clearly defined roles and responsibilities. These elements are intimately linked to their scope of practice, which establishes the boundaries of their professional activities. The scope of practice defines the specific tasks support workers are trained and allowed to perform, ensuring they operate within safe and effective parameters. Consequently, this scope shapes their roles and influences how responsibilities are assigned, reflecting their skills and competencies. For example, support workers within Band 2 roles typically focus on basic patient care tasks, such as assisting with mobility, which are fundamental to daily operations but within a narrower scope of practice. In contrast, those in Band 4 roles may take on more advanced responsibilities, such as facilitating certain therapy exercises or conducting preliminary patient assessments. These responsibilities require a broader scope of practice that includes specific training and competencies.

This sense of “belonging” was described as being fundamental to a support worker feeling valued within their role and as a person. Where they had a clear sense of their identity and their role they demonstrated a strong sense of belonging in the team, but where their identity was unclear or their role and responsibilities were uncertain, they described a disconnect with other members of the team.

#### Identity

Identity was clearly linked to the title that the support worker was afforded; however, this was often unclear or inconsistent in relation to the role they delivered. It was recognised that it was challenging to find a title that suited everybody.

*Even like the assistant is -you are [an] assistant to them*,* but there isn’t. It’s hard to find a title that’s not offensive to anybody (Participant 27)*.

However, the term “assistant” was commonly regarded as being inappropriate and failed to recognise their skills, ability and often vast experience they had working in the role.

being called an assistant had a real sort of negative connotation (participant 24)

There were examples of support workers who had their titles changed informally, allowing them to use titles such as “associate practitioner” or “practitioner” on name badges and emails, but their job descriptions were not changed accordingly. The informality of this led the support workers to question whether it was just to appease them, or whether it was a true recognition of their skills

*Made us feel a little better….but it looks*,* it’s silly*,* but it looks better. On an e-mail…. we say we’re an associate practitioner. It doesn’t sound like assistant (Participant 24).*

Such actions were seen as small token gestures, but also seen as a victory in the “*battle*” they felt they were facing for recognition.

It was extremely evident that the use of terminology when being referred to was key to how they felt as a person and their overarching feeling of value. Frequently support workers described being called “unqualified” compared to the “qualified” registered staff. Extreme cases described how only “qualified” staff were counted in the daily numbers of staff who were working.

*But I know from the old trust that I worked*,* I was very much like they [registered staff] made a point of referring like how many qualified members of staff that they had in*,* and sometimes we weren’t counted in the numbers to say there was only four qualified in (Participant 5)*.

Our participants described this with a deep sadness that such comments significantly undermined their skills and abilities, but also failed to recognise them as a person.

While our participants often described their use of their title negatively, others reported that they liked having the title of support worker as it gave a clear indication of their roles and responsibilities and they had the ability to refer to a registered member of staff. What was evident was a clear desire to have a consistent title that was felt to reflect their abilities, skills and experience and to discourage the use of the term “non-qualified” as often they held multiple qualifications.

Our participants all had experience or were working in NHS settings and all described the historical hierarchy that was associated with this. However, there were many examples of support workers who were working in teams where this hierarchy was less evident and with this brought a real sense that they were a vital part of the team.

Support workers described how they felt a greater sense of belonging when this hierarchy was less evident and felt as though this enabled recognition of their skills rather than the band they were working at.

*There’s no hierarchy. There’s no*,* you’re just a band 4 and I’m a 7*,* you’ve gotta do what I say (Participant 21)*.

Support workers described how the hierarchical attitude was often a learnt attitude that was guided by the management and had a direct impact on the rest of the team. Therefore, the attitude of management was key to how the structure of the team was viewed.

*But I think a lot of that is also set by whoever leads that team and whoever leads that organisation. So if those behaviours are demonstrated at that higher level people below that can see that it’s an OK way to behave (Participant 1).*

The feeling of hierarchy in teams was often confounded by the use of different uniforms for different bands. Support workers often described how they wore a totally different uniform to their registered colleagues which disassociated them from the profession they worked in. Several described how all support workers in the trust wore the same uniform, self-identifying as the “grey army”. It was recognised that where support workers wore the same uniform as their registered colleagues, this feeling of hierarchy was reduced and their belonging increased.

*We all wear the same uniform and we all work together as one team. (Participant 9)*

This was a very visual representation of being part of a profession that support workers appreciated and felt was important in their role and significantly increased their feelings of belonging.

#### Inclusion

A feeling of being included was fundamental to developing a sense of belonging. This related to being included in effective two way communication where they are asked their opinions, but – vitally – these opinions were acted upon.

While communication from managers and within teams varied, it was highlighted that it was central to their role and key to feeling that sense of attachment to a team. Participants shared a continuum of experiences, from not being invited to meetings, to being ignored, to being asked to lead meetings. However, it was evident that having a choice to engage with meetings was fundamental. This culture of inclusivity in meetings promoted a feeling of value. Effective communication and inclusion of everyone in the team highlighted that each role is equally important.

*I think it it’s communicating and you know being inclusive of everybody within that team - everybody’s got a role to play and…every roles is as important as the next persons(Participant 4).*

It was described that many meetings had been stood down during the Covid-19 pandemic due to service pressures, however, this was often just for support workers and registered staff would continue to meet. This generated a feeling of inequity but also caused tension between registered and non-registered staff.

*But the therapy manager came to me and said December’s …. not appropriate time to do meetings bearing in mind just a few days ago*,* every other physios had meetings*,* OTs had meetings*,* they had meetings*,* but all of a sudden because it’s the support workers*,* it’s not an appropriate time for us to have meetings. (Participant 20)*

Where support workers did describe their opinions being sought there was clear evidence that this made them feel their contribution was valuable, however, we had descriptions of where support workers were asked opinions, but no actions would be taken, leading them to describe their opinions being sought as just paying “lip service” to them and made to feel that although they were told they were valued, the lack of action failed to corroborate this.

Good examples involved support workers being asked for their feedback on the performance of other members of staff, being involved in recruitment processes, being asked their opinions by registered staff and being part of conversations about care planning or service development. All of these examples clearly demonstrated to the support worker that their opinions were recognised.

#### Relationships

There was significant variation in how support workers felt as part of the team and consequently how valued they felt. Many support workers felt they were a ‘vital cog’ in the team. However, others reported feeling like ‘just a number,’ often marginalised and underappreciated.

*And I I do think they just think they’re disposable. You know*,* one [support worker] leaves and you get another one in (Participant 21)*.

*When I initially started the role*,* I thought I’ll be with the physio’s*,* occupational therapy. So I initially sat in their office. I’m not part of their team at all*,* I was told (Participant 29)*.

One concerning and negative approach that was reported was the feeling of being “owned” by registered staff. Support workers described how registered staff would often describe them as “my support worker”

*….sometimes really sort of like*,* well*,* you’re my assistant. No*,* no. I’m I’m the trust assistant for this job role*,* but I’m not your assistant. That’s not how this works*,* and that that’s quite degrading (Participant 27)*.

Relationships with close line managers and supervisors was generally reported to be good with examples of them often trying to create development opportunities, but there was evidence that there was a lack of knowledge about what opportunities existed for support workers and how they could access them. Relationships with senior managers was more strained with support workers often reporting that senior management had little or no contact with them and often had poor understanding of their roles.

*When you’ve got your boss saying to you*, *well*, *I don’t really know what you do. You know what I mean (Participant 10)*.

### Empowerment

Empowerment for support workers involves creating an environment where they can exercise agency and make meaningful contributions. Key elements such as opportunities, wellbeing, and support set the stage for empowerment, enabling workers to take ownership of their roles and professional growth. There was a clear pattern in our data suggesting that support workers who were empowered had a greater feeling of being valued. Where they did not feel empowered, there were frequent feelings of frustration and powerlessness.

#### Opportunities

Fair access to opportunities was a key factor in how empowered a support worker felt. Such opportunities included access to training, routes to progress up the bandings as well as having the right support in place.

While many support workers talked about wanting to progress through the bandings – or at least have the choice to be able to, it was very evident that this did not mean they necessarily wanted to become registered AHPs.

*Sometimes there’s a little bit of*,* well*,* obviously you’re only a support worker*,* so you obviously want to be a registered professional. I was a registered professional. And it doesn’t mean I want to be a registered professional again. (Participant 24)*

Some support workers wanted the opportunity to progress beyond Band 4 level which is commonly associated as the ceiling to non-registered workforce. Many cited examples of support workers in other areas who had taken on roles that were Band 5 or higher, however these opportunities were not available in their region or area. Often this led the support worker to feeling they were stuck in the role without the possibility to progress.

*You have that craving for*,* for to get something more and it just. It just feels like it’s a little bit dead end. (Participant 22)*

Many support workers reported being promised promotion, often for years, but frequently these promises were not fulfilled, leading to frustration and disillusionment.

*Yeah. So I’ve done a bit of all sorts really*,* but I’m still on the same banding as when I started*,* there’s been no career progression at all. In 38 years. And that’s it. Yeah*,* it’s been promised. But I’ve never*,* never had it. (Participant 10)*

This participant’s account of being promised promotion, but never receiving it, highlights a key source of frustration for many support workers. A lack of clear and attainable career paths not only limits their growth but can also lead to disillusionment and decreased motivation. This absence of progression opportunities reinforces the feeling of being undervalued and expendable.

There was little knowledge reported relating to the NHS England AHP Support Worker Competency, Education and Career Development Framework [3], with only one of our participants being able to cite this, however, there was knowledge of a perceived increase in the availability of apprenticeships. There were mixed feelings about these with many people having explored them, only to have been told they weren’t eligible, their team could not support them or the courses were not running.

*And I think personally*,* from my job role*,* we did have an apprenticeship and my workplace was supporting it*,* but then the university that we had the apprenticeship with has now stopped running the apprenticeship. So in terms of career development*,* I’m sort of stuck for a little bit just waiting on news. (Participant 25)*

Little other training opportunities were reported to be offered to support workers, with the majority just being mandatory training, in-service training or self-funded courses. There was reported inequity with the availability of funding which was often only offered to registered staff. Often support workers reported that any training they wanted to undertake had to be undertaken in their own time, compared to registered staff being allowed to complete it during working hours. This further increased the feelings of being less important than registered staff.

*I think at the moment there was a budget for qualified staff. I think it was about £1000 each. And you’re just like*,* where’s ours? I don’t like it when I get copied into those emails…So yeah*,* as far as I’m aware*,* funding wise at the moment*,* there hasn’t been any funding. (Participant 21)*

#### Wellbeing

Our participants reported how their physical and mental wellbeing was key to feeling empowered. Where their physical and mental health was considered and promoted, they were more able to seek opportunities and had greater confidence to explore progression opportunities. While they felt their wellbeing was deemed important by direct supervisors and their teams, there was a feeling that more senior management neglected to consider this.

*We do care about people*,* about our patients*,* but the management doesn’t care about their employee*,* especially when it comes to our health*,* our mental health*,* physical health. Literally don’t care about us at all. (Participant 28)*

“Burn-out” was discussed by several of our participants and related to a reported lack of consideration of their wellbeing.

*I think a lot of them get burnt out because they’re [support workers]. going onto the wards – they’re doing their job*,* they’re trying to learn more. They’re trying to maybe sometimes they’re trying to even progress*,* but there are barriers being put in the way. (Participant 20)*

A sense of poor wellbeing and a poor work life-balance for some of our participants led them to feel that they would look to leave their current roles. However, many had remained loyal for many years – despite ongoing feelings of frustration.

*Some days are good*,* some days are bad. I’m at the breaking point now. And I’m saying*,* how much can I put this smile on before I give up and just walk away? (Participant 29)*

Physical wellbeing was also reported to be important. This involved being allowed time to attend medical appointments as well as ensuring that manual handling practices were safe for them to undertake.

#### Support

Feeling supported enhanced the sense of empowerment of our participants. This was discussed as being formal support such as supervision and annual appraisals, but also the support that they received from their teams by means of moral support and encouragement.

Appraisals were often reported to be ineffective in helping support their development, despite managers and supervisors affording time to complete them.

*I struggle with appraisals. I do think they’re a tick box exercise. I’ve been in the overachieving box for as long as I can remember. And who cares if you achieve your objectives*,* or if you don’t*,* they [management] don’t. Nobody…. my mandatory training is always up to date*,* so apart from that I don’t think anyone cares. I don’t find it helpful. (Participant 21)*

When explored further, it was evident that appraisals often linked to objectives for the team rather than objectives for the person. This was particularly evident when a support worker had been in the same role for many years as it became apparent that setting new personal development objectives was often just duplicating previous years.

There was evidence that the amount of support had reduced during the Covid-19 pandemic with supervisions, appraisals and training all cancelled. The extent to which this had been increased again varied in organisations, but it was key that support workers felt supported – and thus empowered in order to feel that they had value.

Access to learning and development opportunities fosters empowerment by allowing support workers to build confidence and advance their skills. Prioritising wellbeing—both physical and mental—provides a foundation for resilience, enabling workers to engage more fully with empowerment opportunities. Additionally, supportive feedback from supervisors, coupled with trust to make independent decisions, strengthens their sense of autonomy and agency. While empowerment contributes to feeling valued, they are distinct experiences. Support workers may feel valued when recognised and included, but empowerment goes further, giving them control over their roles and allowing them to actively shape their work environment. Empowerment, therefore, complements belonging and recognition by enabling workers not only to feel appreciated but also to exert influence in their roles.

## Discussion

The aim of this study was to undertake an exploration of the feelings of “value” that AHP support workers associate with their role. We interviewed AHP support workers with a wide range of experience and roles across England and found that there are key factors which contribute to a feeling of being valued. Whilst the 2013 Cavendish Report [4] highlighted the fact that support workers did not feel valued, this is the first study to the authors knowledge that has explored the reasons for this and sought to gain an understanding of the factors that contribute to the feeling of being valued. Based on our findings, it appears that for support workers to feel valued, they must experience empowerment, a strong sense of belonging, and acknowledgment of their skills and expertise. These prerequisites for feeling valued closely mirror Maslow’s hierarchy of needs [14], which asserts that individuals are driven by five fundamental categories of needs: physiological, safety, love, esteem, and self-actualisation. Therefore, we propose that feeling valued and being motivated are intrinsically linked and constitute a fundamental human need. Support workers who exhibited high levels of motivation typically reported feeling highly valued in their role. Conversely, those who did not perceive themselves as valued tended to exhibit markedly lower levels of motivation to fulfil their responsibilities. Furthermore, when they lacked a sense of value, both their physical and mental well-being were adversely affected.

Our findings align with broader research indicating that job titles and terminology significantly influence professional identity, perceived value, and inclusivity. For example, research in organisational psychology has shown that titles can shape both self-perception and others’ perceptions, affecting motivation and workplace engagement [9]. In healthcare settings, similar studies highlight how hierarchical language can reinforce traditional power dynamics, potentially marginalising non-registered or support roles. Similar to our findings, Kessler et al. [15] found that healthcare assistants often felt undervalued due to being labelled as ‘unqualified,’ which impacted their professional self-esteem and sense of belonging within clinical teams.

Additionally, research by Nancarrow et al. [10] emphasises the importance of clear role delineations and appropriate terminology for fostering a sense of professional identity among support workers in intermediate care. Similarly, evidence suggest that ambiguous language surrounding support roles can lead to confusion over responsibilities and hinder effective delegation, ultimately affecting the perceived value of these roles within healthcare teams [16].

A study of the role of Australian Allied Health Assistants highlighted the significance of relationships, role delineations, professional identity, and access to training [12]. Emphasising the necessity of a defined scope of practice, they underscored the importance of establishing clear competencies and scopes of practice. This finding aligns with our own observations, as our data revealed that a well-defined scope of practice and boundaries provided crucial support for AHP support workers in carrying out their responsibilities, fostering a sense of value and recognition in their roles. Evidence to support delegation is lacking and inconsistent [16], which has the potential to lead to lack of understanding about what can be safely delegated and therefore who has responsibility.

The relationship between registered and non-registered staff emerged as a critical theme, particularly with the perception of support workers being “owned” by registered professionals. This dynamic is often tied to the delegation of tasks, where registered professionals retain ultimate accountability. The language used by registered staff, implying ownership over tasks and, by extension, over the support workers themselves, can negatively impact the support workers’ self-efficacy. Studies on healthcare delegation indicate that, while delegation is essential for efficient care delivery, it is often accompanied by a lack of clarity on what tasks can be safely and effectively delegated [10]. When role boundaries and task responsibilities are not well-defined, support workers may feel diminished, as they are seen merely as extensions of registered professionals rather than autonomous contributors. This aligns with Nancarrow’s [10] findings, which suggest that inconsistent delegation practices contribute to confusion regarding accountability and can impact the working relationship between registered and non-registered staff.

The use of hierarchical language, such as “qualified” and “unqualified,” further reinforces this dynamic. Such terminology suggests a lack of value or capability on the part of support workers, despite the essential role they play in patient care. The distinction between “qualified” and “unqualified” also implies that support workers lack qualifications entirely, which undermines their professional identity and contributes to a sense of inferiority. Research has shown that language shapes workplace culture and influences perceptions of professional status and self-worth [17]. Additionally, the wording in studies can create inherent biases. For instance, a discrete choice experiment that asked participants to choose between treatment from a “fully qualified occupational therapist or physiotherapist” versus an assistant implicitly conveyed that assistants were less capable, regardless of their actual qualifications and competencies [18]. This language not only skews participant perceptions but also reinforces the bias that support workers are less skilled or knowledgeable.

Therefore, it is crucial to exercise caution in both clinical and research settings regarding the terminology used to describe roles. Language that respects the expertise and contributions of support workers can help to enhance their sense of self-efficacy and professional identity, ultimately leading to better team dynamics and patient care outcomes. As research by MacLeod and Clarke [19] illustrates, inclusive language fosters a sense of value and belonging among workers, which is essential for building a cohesive healthcare team. By carefully considering the language used to describe registered and non-registered roles, healthcare organisations can promote a more respectful and inclusive environment that recognises the full scope of each role.

An unexpected, but commonly reported, issue was the reported use of the term “just a support worker”. This was a term used by support workers themselves often but was reported to have been developed from historical attitudes and ways that support workers had been termed over the years. The detrimental effects of using this term were evident with support workers feeling of less value than their registered colleagues. Evidence suggests that the way that employees are spoken to has a direct impact on their overall experience of their role as well as their level of engagement with their work [9], which was evident from our data.

To ensure support workers feel valued, healthcare organisations should implement recognition practices that acknowledge their contributions. These could include regular feedback, celebrating achievements, and involving support workers in decision-making processes. Creating accessible career development pathways, such as training programs or progression routes within the NHS banding system, can also empower support workers and enhance their sense of agency. According to Nancarrow et al. [10], when employees have a clear path for advancement, they are more likely to stay committed and feel a sense of purpose in their roles.

### Strengths and limitations

This study embraced diversity by including participants with varying characteristics, such as their profession within the AHP field, area of work, location, and levels of experience. Our aim was to capture a comprehensive range of experiences within the AHP support worker community. To ensure the study’s relevance and effectiveness, we actively involved a stakeholder group in the study’s development, interpretation, and presentation of results. Although this study focused on AHP support workers, there may be similarities with nursing support workers, which were not explored in this research. Despite the breadth of AHP professions, we acknowledge limitations in our sample diversity, recruiting participants from only seven out of fourteen unique AHP professions. However, the use of AHP support workers in the other AHP professions is less sizable in the NHS such as those, for example, in music therapy. Moving forward, efforts to broaden participation across all AHP disciplines could provide deeper insights into the challenges and experiences of support workers in diverse healthcare settings.

## Conclusion

The contributions of AHP support workers highlight the critical importance of recognising their multifaceted roles within the NHS healthcare system. Despite facing numerous challenges, these dedicated, often highly skilled professionals frequently prioritise patient care above their own interests. However, it is imperative to acknowledge that to foster a supportive and effective healthcare environment, AHP support workers must feel recognised, empowered, and included in their teams. Recognising and appreciating their unique skills and providing opportunities for ongoing learning and professional development are essential steps towards ensuring their well-being and fostering a culture of inclusivity within the healthcare community. By affirming the significance of AHP support workers and actively supporting their growth and development, we not only enhance the quality of patient care but also cultivate a more resilient and empowered healthcare workforce.

## Supplementary Information

Supplementary Material 1.

Supplementary Material 2.

## Data Availability

The datasets used and/or analysed during the current study are available from the corresponding author on reasonable request.

## Abbreviations

AHA: Allied Health Assistant
AHP: Allied Health Profession
NHS: National Health Service
EOI: Expression of Interest
