# Supplementary material for: Feeling valued at work: a qualitative exploration of allied health profession support workers
Source: BMC Health Serv Res. 2024 Nov 29;24:1511. doi: 10.1186/s12913-024-11879-z (PMC11607816; doi:10.1186/s12913-024-11879-z)
Supplement: Supplementary file 2 — Supplementary Material 2. [file 12913_2024_11879_MOESM2_ESM.docx]

**Topic guide**

| **Background**  **“Please can you tell me a little about your career”** | Career synopsis  Reasons for entering profession  Career development throughout career  Future career plans |
| --- | --- |
| Current role  “I’d like to hear more about your current role and what you enjoy and what things are less enjoyable”  **SETTING** | Relationship with registered staff  Opportunities for development  Relationships with managers/organisation  Relationship with patients  What could be better?  What is good about role?  CONFIDENCE TO UNDERTAKE ROLE |
| Value  “In your role, how valued do you feel?” What factors influence this? | By patients  Team  Profession  Organisation  NHS  Union/Professional body |
| Changes to the NHS  “Since you’ve worked for the NHS have you experienced any large changes in your role?” | Attitude towards changes  Involved in?  Experiences  Understanding of IC and rehab frameworks |
| Training  “What sort of training do you get or would you like to have?” | IST?  HOW DO YOU SET OBJECTIVES WHEN/IF YOU REACH THE TOP OF YOUR BAND?  PDR  External training  Reasons for doing/not-doing? |
| Policy  “Are you aware of any national policies or strategies related to support workers?” What affect might these have? | Awareness of SW development  CoP?  Framework? |
| Future plans  “Do you see your current role changing at all? How do you feel about this?” | Any thoughts about leaving?  Developing role |
